# Evaluation of the novel folate receptor ligand [^18^F]fluoro-PEG-folate for macrophage targeting in a rat model of arthritis

**DOI:** 10.1186/ar4191

**Published:** 2013-03-01

**Authors:** Yoony YJ Gent, Karin Weijers, Carla FM Molthoff, Albert D Windhorst, Marc C Huisman, Desirée EC Smith, Sumith A Kularatne, Gerrit Jansen, Philip S Low, Adriaan A Lammertsma, Conny J van der Laken

**Affiliations:** 1 Department of Rheumatology, VU University Medical Center, De Boelelaan 1117, Amsterdam, 1081 HV, The Netherlands; 2 Department of Radiology & Nuclear Medicine, VU University Medical Center, De Boelelaan 1117, Amsterdam, 1081 HV, The Netherlands; 3 Department of Clinical Chemistry, VU University Medical Center, De Boelelaan 1117, Amsterdam, 1081 HV, The Netherlands; 4 Department of Chemistry, Purdue University, 560 Oval Drive, West Lafayette, IN 47907, USA

## Abstract

**Introduction:**

Detection of (subclinical) synovitis is relevant for both early diagnosis and monitoring of therapy of rheumatoid arthritis (RA). Previously, the potential of imaging (sub)clinical arthritis was demonstrated by targeting the translocator protein in activated macrophages using *(R)*-[^11^C]PK11195 and positron emission tomography (PET). Images, however, also showed significant peri-articular background activity. The folate receptor (FR)-β is a potential alternative target for imaging activated macrophages. Therefore, the PET tracer [^18^F]fluoro-PEG-folate was synthesized and evaluated in both *in vitro *and *ex vivo *studies using a methylated BSA induced arthritis model.

**Methods:**

[^18^F]fluoro-PEG-folate was synthesized in a two-step procedure. Relative binding affinities of non-radioactive fluoro-PEG-folate, folic acid and naturally circulating 5-methyltetrahydrofolate (5-Me-THF) to FR were determined using KB cells with high expression of FR. Both *in vivo *[^18^F]fluoro-PEG-folate PET and *ex vivo *tissue distribution studies were performed in arthritic and normal rats and results were compared with those of the established macrophage tracer *(R)*-[^11^C]PK11195.

**Results:**

[^18^F]fluoro-PEG-folate was synthesized with a purity >97%, a yield of 300 to 1,700 MBq and a specific activity between 40 and 70 GBq/µmol. Relative *in vitro *binding affinity for FR of F-PEG-folate was 1.8-fold lower than that of folic acid, but 3-fold higher than that of 5-Me-THF. In the rat model, [^18^F]fluoro-PEG-folate uptake in arthritic knees was increased compared with both contralateral knees and knees of normal rats. Uptake in arthritic knees could be blocked by an excess of glucosamine-folate, consistent with [^18^F]fluoro-PEG-folate being specifically bound to FR. Arthritic knee-to-bone and arthritic knee-to-blood ratios of [^18^F]fluoro-PEG-folate were increased compared with those of *(R)-*[^11^C]PK11195. Reduction of 5-Me-THF levels in rat plasma to those mimicking human levels increased absolute [^18^F]fluoro-PEG-folate uptake in arthritic joints, but without improving target-to-background ratios.

**Conclusions:**

The novel PET tracer [^18^F]fluoro-PEG-folate, designed to target FR on activated macrophages provided improved contrast in a rat model of arthritis compared with the accepted macrophage tracer *(R)-*[^11^C]PK11195. These results warrant further exploration of [^18^F]fluoro-PEG-folate as a putative PET tracer for imaging (sub)clinical arthritis in RA patients.

## Introduction

Activated macrophages infiltrate inflamed synovium of rheumatoid arthritis (RA) patients in early stages of the disease and play a pivotal role in sustaining the chronic phase of RA [1,2]. Assessment of these synovial macrophages could provide a means to predict and monitor clinical disease activity, since the number of synovial macrophages correlates with disease activity scores and response to treatment [1].

In a large subset of RA patients, clinical manifestations of disease are preceded by a preclinical phase characterized by the presence of auto-immune antibodies and presumably subclinical synovitis [3,4]. Visualization of macrophages in subclinically inflamed synovium may thus enable earlier initiation of therapy, which could prevent the onset of joint damage. Moreover, RA patients in remission may also benefit from detection of subclinical synovitis, since joint damage has been noticed in a substantial subset of these patients despite clinically quiescent disease [5].

Advanced imaging techniques, such as positron emission tomography (PET), enable non-invasive visualization of macrophages, but require availability of suitable radioligands. Previously, feasibility of imaging (sub)clinical arthritis was demonstrated using the macrophage targeting PET tracer *(R)*-[^11^C]PK11195 (1-[2-chlorophenyl]-*N*-methyl-*N*-[1-methyl-propyl]-3-isoquinoline carboxamide), that binds to the translocator protein that is up-regulated in activated macrophages [6]. Due to background uptake of this tracer in peri-articular tissue, however, detection of subclinical synovitis may be suboptimal [6,7]. Consequently, there is a need for other macrophage tracers with lower background binding in peri-articular tissues.

A promising alternative target present on macrophages is the folate receptor (FR), to which both folic acid and 5-methyltetrahydrofolate (5-Me-THF) bind with high (nanomolar) affinity [8]. In addition, the number of normal tissues expressing FR is limited [9,10], and overexpression has only been found on activated macrophages and several (epithelial) cancer cells [8,9,11,12]. Hence, the FR has been recognized as an attractive molecular target for diagnostic as well as therapeutic approaches [12-18].

Xia *et al*. [11] demonstrated that, in contrast to resting macrophages, activated macrophages at sites of inflammation and infection express a functional β-isoform of FR. In addition, it was shown that FR-β is expressed abundantly on macrophages in synovial tissue of (clinically active) RA patients, providing opportunities for targeting this disease with highly selective PET tracers and folate antagonist drugs [12,13,15].

To date, various imaging procedures for targeting FR have been developed [17-19]. Gamma scintigraphic imaging with EC20, a ^99m^Tc-labelled folate agent, has been successfully applied for imaging of arthritis and infection in preclinical studies and detection of arthritic joints in RA patients [20-22]. As PET provides higher sensitivity and spatial resolution, it is particularly interesting for detection of subclinical arthritis.

In the present study, a novel FR targeting fluorine-18 labelled PET tracer, [^18^F]fluoro-polyethylene glycol (PEG)-folate was developed. Several ^18^F-based folate tracers have been synthesized thus far [23-26], but [^18^F]fluoro-PEG-folate was anticipated to show improved kinetics because of the introduction of the PEG moiety [27]. Therefore, its characteristics were assessed in *in vitro *FR binding studies and in *ex vivo *tissue distribution and *in vivo *PET studies using a methylated bovine serum albumin (mBSA) induced arthritis model in the rat.

## Materials and methods

### Synthesis of [^18^F]fluoro-PEG-folate and (R)-[^11^C]PK11195

**Figure 1:**
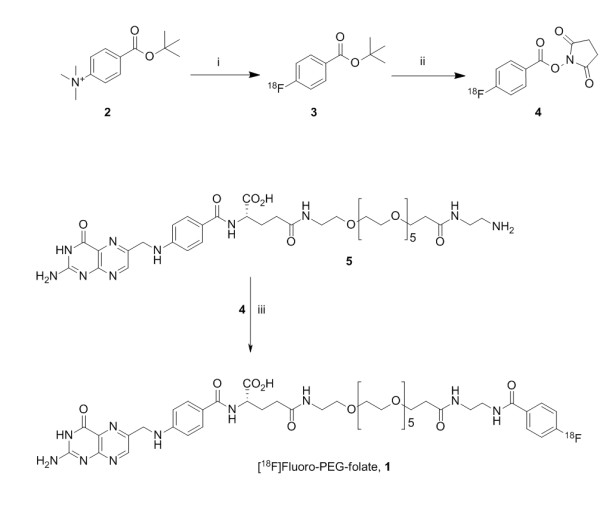
**Synthesis of [^18^F]fluoro-PEG-folate**. Synthesis of [^18^F]fluoro-PEG-folate. Conditions: ***i***: [^18^F]fluoride, acetonitrile, K[2.2.2], K_2_CO_3_, 90°C, 10 minutes; ***ii***: a: tetrapropylammonium hydroxide, b: tetramethyl-o-(N-succinimidyl)uranium tetrafluoroborate, 30 to 50% (for *i *and *ii*); ***iii***: 150 mM borate buffer, 30 minutes ambient temperature, 70 to 90%.

[^18^F]fluoro-PEG-folate (**1**) was synthesized in a two-step procedure (Figure 1): First, [^18^F]succinylfluorobenzoate ([^18^F]SFB, **4**) was obtained by fluorination of the SFB precursor (**2**) (ABX, Radeberg, Germany). The precursor (5 mg) was dissolved in 1 mL acetonitrile and, after standard work-up of the [^18^F]fluoride solution, the precursor was added and the reaction was allowed to proceed for 10 minutes at 90°C. The labeled precursor (**3**) was then deprotected with 40 µL of tetrapropylammonium hydroxide, and the reaction mixture was evaporated and treated with tetramethyl-o-(N-succinimidyl)uranium tetrafluoroborate to generate [^18^F]SFB (**4**). This [^18^F]SFB solution was passed over a preconditioned (10 mL sterile ethanol 96% and then 10 mL water for injection) Waters Sep-Pak C18 cartridge (Milford, MA, USA). After washing with 10 mL 15% acetonitrile in water for injection and subsequent elution of the product from the cartridge with 1.5 mL acetonitrile, the [^18^F]SFB solution was evaporated to a volume of approximately 100 µL. A solution of 2 mg PEG-folate precursor (**5**) in 1 mL borate buffer (150 mM, pH 8.6) was added to the 100 µL solution of [^18^F]SFB (**4**) in acetonitrile and reacted for 30 minutes at ambient temperature. The reaction mixture was injected onto a Gemini 5µ C18 HPLC column (Phenomenex, Torrance, CA, USA), which was eluted with an 80/20 mixture of acetonitrile/0.1% trifluoroacetic acid in water at 3.0 mL/minute and UV at 280 nm. The product, [^18^F]fluoro-PEG-folate, eluted at 31 to 33 minutes. This fraction was collected in 30 mL water and passed over a preconditioned (10 mL sterile ethanol 96% and then 10 mL water for injection) Waters Sep-Pak tC18 (Milford, MA, USA). After washing with 20 mL water for injection, the product was eluted from the cartridge with 1.0 mL sterile ethanol (96%) and 14 mL of a sterile and pyrogen free sodium phosphate solution (7.09 mM, pH 5.4) in saline. The final mixture was passed over a Millex GV 0.22 µm filter (Millipore, Billerica, MA, USA), yielding a sterile, isotonic and pyrogen free solution of 0.5-1.5 GBq of [^18^F]fluoro-PEG-folate. The product was analyzed using HPLC on a Varian ChromSpher 5 C18 column (Lake Forest, CA, USA), which was eluted with a 20/80 mixture of acetonitrile/0.1% trifluoroacetic acid in water at 1.2 mL/minute and UV at 280 nm.

*(R)-*[^11^C]PK11195 was synthesized routinely with a radiochemical purity >98% and mean specific activity of 95.7 ± 28.4 GBq/μmol, as described previously [28].

### In vitro FR binding competition assay

*In vitro *FR binding competition assays were performed essentially as described previously [12]. In short, FR expressing human KB cells grown in folate deficient RPMI medium (Buffalo, NY, USA) were detached by trypsinization and washed twice in ice-cold Hepes-buffered saline solution (pH 7.4) at a density of 1 × 10^6 ^cells/mL. Next, 100 µL of this cell suspension was incubated with folate-fluorescein isothiocyanate (FITC) [11] to a final concentration of 10 nM in the absence or presence of a 0.1- to 100-fold molar excess of unlabelled folic acid, 5-Me-THF (Merck Eprova, Schaffhausen, Switzerland) or unlabelled, non-radioactive reference compound fluoro-PEG-folate. Following 15 minutes of incubation at 4#176;C, cells were centrifuged and analyzed for displacement of folate-FITC binding by flow cytometric analysis (FACS Scan, BD Biosciences, San Jose, CA, USA).

### Animals

Wistar rats (male, body weight approximately 200 to 300 g, Charles River International Inc., Sulzfeld, Germany) were provided with standard food (16% protein rodent diet, Harlan Laboratories Inc., Madison, WI, USA) and water *ad libitum*. Rats were housed in groups of four in conventional cages kept in a room with a 12-hour light/dark cycle and constant room temperature (21ºC) and humidity level (50%). For experiments designed to reduce plasma folate levels, arthritic rats were housed in cages with a grid floor to prevent folate intake from feces and were fed *ad libitum *with folate deficient chow (Arie Blok BV, Woerden, The Netherlands).

All animal experiments were performed in accordance with the Dutch law on animal experimentation and were approved by the committee on animal experimentation of the VU Medical Center.

### Induction of knee arthritis and study timelines

Antigen induced arthritis was generated as described previously [29] with adaptations for optimal PET imaging of arthritis. Briefly, rats were immunized subcutaneously at Days 0 and 7 with 200 μL of a solution of 50 mg mBSA (Sigma-Aldrich Chemie BV, Zwijndrecht, The Netherlands) dissolved in 1 mL 0.9% NaCl and emulsified in 1 mL complete Freund's adjuvant (Sigma-Aldrich, Steinheim, Germany) and 1 mL custom Bordetella pertussis antigen (Becton Dickinson, Breda, The Netherlands). To induce local arthritis (Day 20), rats received an intra-articular injection in the right knee with 60 μL of a solution of 10 mg mBSA in 1 mL 0.9% NaCl. At Day 27, PET scans were performed. Immediately after PET scanning, rats were sacrificed and *ex vivo *tissue distribution of the tracers was measured.

### PET scanning protocol

Scans were performed using a double LSO/LYSO layer ECAT High Resolution Research Tomograph (HRRT, CTI/Siemens, Knoxville, TN, USA), a small animal and human brain 3-dimensional (3D) scanner with high spatial resolution (2.3 to 3.4 mm full width at half maximum) and high sensitivity [30]. Arthritic rats (*n *= 4) or normal rats (*n *= 7) were anesthetized (approximately 2% isoflurane with an oxygen flow of 0.6 to 0.8 L/min) and positioned in the HRRT. A static transmission scan (using a rotating 740 MBq ^137^Cs point source) of six minutes duration was performed and following intravenous injection of 15.1 ± 3.2 MBq [^18^F]fluoro-PEG-folate via a jugular vein canula, an emission scan was acquired for one hour. PET data were normalized and corrected for scatter, randoms, attenuation, decay and dead time. Data were converted into 16 sinograms (5 × 5, 5 × 10, 3 × 15, 2 × 30, 2 × 60, 2 × 150, 2 × 300, 1 × 600, 2 × 900 s). Images were reconstructed using an iterative 3D ordinary Poisson ordered-subsets expectation-maximization algorithm with 8 iterations and 16 subsets. Resulting images had a matrix size of 256 × 256 × 207 voxels, each with a dimension of 1.21 × 1.21 × 1.21 mm^3^.

### PET image analysis

PET images were processed using AMIDE software (Amide's a Medical Image Data Examiner, version 0.9.2) [31]. Ellipsoid regions of interest (ROI) (6 × 17.7 × 7.4 mm^3^) were manually drawn over left and right knees. ROIs were projected onto the dynamic image sequence, thereby generating time-activity curves, expressed as standardized uptake values (SUV), where SUV represents mean ROI radioactivity concentration normalized for injected dose and body weight. For determining [^18^F]fluoro-PEG-folate uptake in both knees, SUV was averaged over the period from 50 to 60 minutes post-injection. Blood concentrations, also expressed as SUV, were obtained from manually drawn ROIs (4 planes, 12 voxels/plane) in the heart. The image derived injected dose (IDID) was obtained from a ROI covering the whole rat.

### Ex vivo tissue distribution studies

Sixty minutes after intravenous injection of 15.1 ± 3.2 MBq [^18^F]fluoro-PEG-folate or 10.5 ± 2.9 MBq *(R)-*[^11^C]PK11195, blood was collected from anesthetized rats by heart puncture. Subsequently, rats were sacrificed by cervical dislocation. Both knees (*in toto*), bone obtained from the right hind leg, and blood and tissue from various internal organs were excised and weighed. Radioactivity in each tissue (in percentage injected dose per gram of tissue; %ID/g) was determined using an LKB 1282 Compugamma CS gamma counter (LKB Wallac, Turku, Finland). Tissue distribution studies were performed in four arthritic rats and eight normal rats for [^18^F]fluoro-PEG-folate and in five arthritic rats for *(R)-*[^11^C]PK11195.

### Histopathology and immunohistochemistry

Both knees were dissected *in toto*, fixed (10% paraformaldehyde, 2% sucrose/phosphate buffered saline, pH 7.3) for seven days at 4ºC and decalcified in 122.8 mM sodium ethylenediaminetetraacetic acid (Na_2_-EDTA.2H_2_O) (Merck, Darmstadt, Germany) and 112.5 mM sodium hydroxide (NaOH) (pH 7.2), (Sigma-Aldrich Chemie BV, Zwijndrecht, The Netherlands) for approximately seven weeks at 4ºC. After rinsing, knees were processed for paraffin embedding. Sagittal sections (5 µm) from the center of the joint were used for immunohistochemical staining of macrophages with a mouse anti-rat monoclonal antibody ED1 (MCA341R, Serotec, Düsseldorf, Germany), that recognizes the rat homologue of human CD68 [32]. Briefly, after antigen retrieval with a solution of 0.1% pepsin (Sigma-Aldrich Chemie BV, Zwijndrecht, The Netherlands), 0.1% of HCl 37% in phosphate buffered saline (PBS) per slide for 30 minutes at 37ºC, sections were incubated for 1 hour with 1:100 diluted ED1 antibody in 0.1% BSA/PBS. The detection EnVision™ kit (K4063 dual-link-HRP rabbit/mouse, DAKO, Glostrup, Denmark) was used according to the instructions of the manufacturer. After washing with PBS, slides were stained for peroxidase activity with 3.3'-diaminobenzidine tetrahydrochloride containing 0.01% H_2_O_2_. Subsequently, sections were counterstained with hematoxylin, dehydrated and mounted. Negative controls were included by replacement of the primary antibody with 1% BSA/PBS. Images were captured by using a Leica 4000B microscope and Leica digital camera DC500 (Microsystems B.V., Rijswijk, The Netherlands).

### In vivo blockade of FR

In order to investigate whether [^18^F]fluoro-PEG-folate binds specifically to FR, blocking studies were performed in rats (*n *= 6) by pre-administration of a ≥100-fold molar excess of the folate conjugate glucosamine-folate five minutes prior to the injection of [^18^F]fluoro-PEG-folate. Glucosamine-folate was preferred over folic acid because of its increased solubility at low pH, preventing precipitation in the kidneys [33]. PET and *ex vivo *tissue distribution studies were performed as described above.

### Studies of rats with reduced plasma folate levels

Conceptually, high plasma and tissue levels of 5-Me-THF, the naturally occurring form of folate, in rodents (approximately 10-fold higher than in humans [34]) may compete with [^18^F]fluoro-PEG-folate for binding to FR. To investigate this, one subgroup of rats (*n *= 4) was fed with folate deficient chow (Arie Blok BV, Woerden, The Netherlands) for 14 days. Plasma 5-Me-THF levels were determined by positive electrospray liquid chromatography tandem mass spectrometry (LC-MS/MS) (Applied Biosystems, Foster City, CA, USA) with a plasma intra-assay CV <4% and inter-assay <2% [35].

### Statistical analysis

Statistical tests were performed using SPSS version 15.0 for Windows (SPSS Inc., Chicago, IL, USA). A Wilcoxon signed rank (exact) test was used to determine differences in paired observations, for example, uptake of [^18^F]fluoro-PEG-folate in the arthritic versus contralateral knee. Mann-Whitney (exact) tests were performed to analyze differences in (a) [^18^F]fluoro-PEG-folate uptake in arthritic versus normal knee, (b) [^18^F]fluoro-PEG-folate versus *(R)-*[^11^C]PK11195 uptake, (c) [^18^F]fluoro-PEG-folate uptake in the arthritic knee under normal versus folate deficient conditions, and (d) tissue uptake ratios. A *P*-value <0.05 was considered as statistically significant. Results are presented as mean ± standard deviation (SD), unless stated otherwise.

## Results

### Synthesis of [^18^F]fluoro-PEG-folate

[^18^F]fluoro-PEG-folate was synthesized via a two-step radiochemical reaction sequence (Figure 1). The intermediate [^18^F]SFB was obtained in 30 to 50% decay corrected radiochemical yield, while the reaction with **3 **was performed in 70 to 90% decay corrected radiochemical yield (Figure 1). Representative HPLC chromatograms are depicted in Additional file 1, Figure S1. The procedure yielded 300 to 1,700 MBq of [^18^F]fluoro-PEG-folate with a radiochemical purity >97% and a specific activity of 40 to 70 GBq/µmol at the end of synthesis.

### In vitro folate competition assay

**Figure 2:**
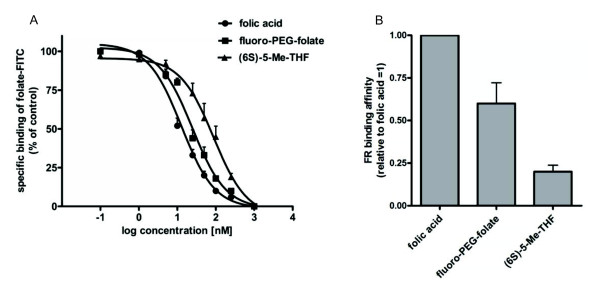
**FR-binding affinities**. (**A**) Displacement of folate-FITC binding from human KB cells (10 nM, 4ºC) with increasing concentrations of unlabelled folic acid, fluoro-PEG-folate and 5-Me-THF. (**B**) Relative binding affinities to FR of folic acid (set at 1) versus fluoro-PEG-folate and 5-Me-THF. Results are presented in mean ± SEM of seven separate experiments.

*In vitro *binding competition assays with the FR expressing KB cell line were performed to compare relative binding affinities of fluoro-PEG-folate with that of folic acid and the biologically active and circulating plasma form of folate, 5-Me-THF. Figure 2A shows displacement of folate-FITC from the receptor in the presence of increasing concentrations of folic acid, unlabelled fluoro-PEG-folate and 5-Me-THF. Relative binding affinities were calculated from these studies indicating that binding affinity for FR of fluoro-PEG-folate is 1.8-fold lower than that of folic acid, but 3-fold higher than that of 5-Me-THF (Figure 2B).

### Presence of synovial macrophages in arthritic knees

**Figure 3:**
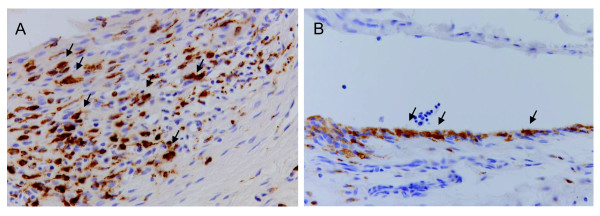
**Immunohistochemical staining for macrophages**. Tissue sections (x200 magnification) of (**A**) arthritic and (**B**) contralateral knees with anti-rat CD68 (ED1) staining (brown staining) for synovial macrophages. Arrows indicate examples of ED1 positive macrophages.

Arthritis induced by intra-articular injection of mBSA in the knees of immunized rats was characterized by influx of macrophages in the synovium as shown by immunohistochemical staining (Figure 3A). In contrast, in the contralateral control knee, macrophage infiltration was minimal compared with the arthritic knee (Figure 3B).

### PET studies

**Figure 4:**
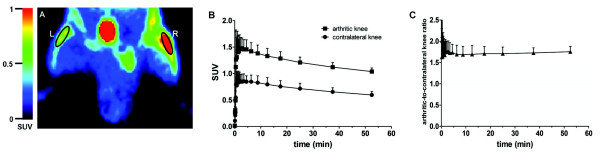
**[^18^F]fluoro-PEG-folate images and corresponding time-activity curves of arthritic and normal knees**. (**A**) Example of [^18^F]fluoro-PEG-folate image of arthritic rat at the level of the knees (L = left contralateral knee, R = right arthritic knee). (**B**) corresponding time-activity curves (of ROI as indicated in 4A) of arthritic and contralateral knees. (**C**) Arthritic-to-contralateral knee ratio as function of time. Results are presented as mean ± SD of four arthritic rats.

[^18^F]fluoro-PEG-folate images clearly visualized arthritis (Figure 4A). SUV of [^18^F]fluoro-PEG-folate in arthritic knees was markedly increased compared with that in contralateral control knees (1.04 ± 0.06 vs 0.60 ± 0.06, respectively) (Figure 4A) and in (right) knees of normal rats (0.67 ± 0.07, *P *<0.01).

Knee time-activity curves of ROIs of arthritic knees and contralateral knees showed that increased [^18^F]fluoro-PEG-folate uptake in the arthritic knee was evident rapidly after injection of the tracer and persisted for one hour (Figure 4B), with a slightly increasing arthritic-to-contralateral knee ratio over time (Figure 4C).

Blood [^18^F]fluoro-PEG-folate levels peaked in the first minutes, but decreased within two minutes below the level of the arthritic knee (see Additional file 2, Figure S2A). The arthritic knee-to-blood ratio increased over time, pointing to retention of the tracer in the arthritic joint in the presence of clearance from the blood (see Additional file 2, Figure S2B).

### Ex vivo tissue distribution studies

**Figure 5:**
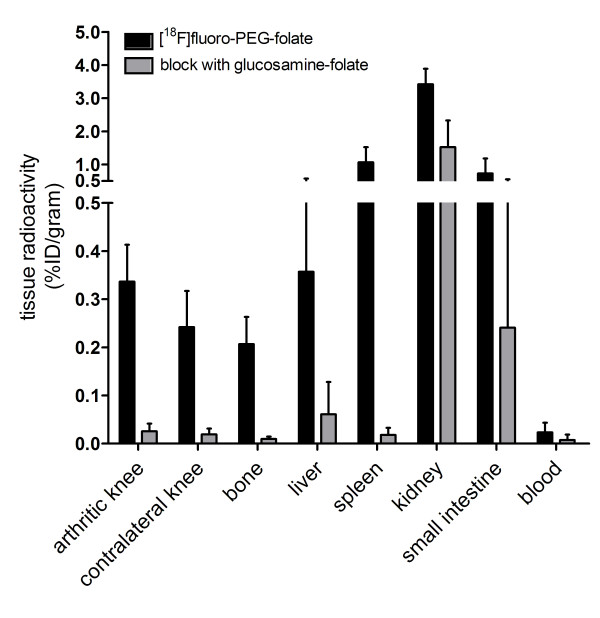
**Tissue distribution of [^18^F]fluoro-PEG-folate**. Tissue distribution of [^18^F]fluoro-PEG-folate in arthritic rats without (black bars) and with (gray bars) pre-treatment with a ≥100-fold molar excess of glucosamine-folate. Results are presented as mean ± SD of four untreated and four pre-treated rats.

[^18^F]fluoro-PEG-folate uptake (expressed as mean %ID/g) in arthritic knees was increased compared with contralateral knees (0.34 ± 0.08 vs 0.24 ± 0.08, respectively) (Figure 5) and (right) knees of normal rats (0.16 ± 0.04, *P *<0.01), thereby confirming the PET data.

[^18^F]fluoro-PEG-folate uptake was also observed in the liver (0.36 ± 0.22), spleen (1.07 ± 0.45), intestine (0.72 ± 0.45) and kidneys (3.42 ± 0.47) (Figure 5). These observations were in line with the accumulation seen in PET images (see Additional file 3, Figure S3A).

**Figure 6:**
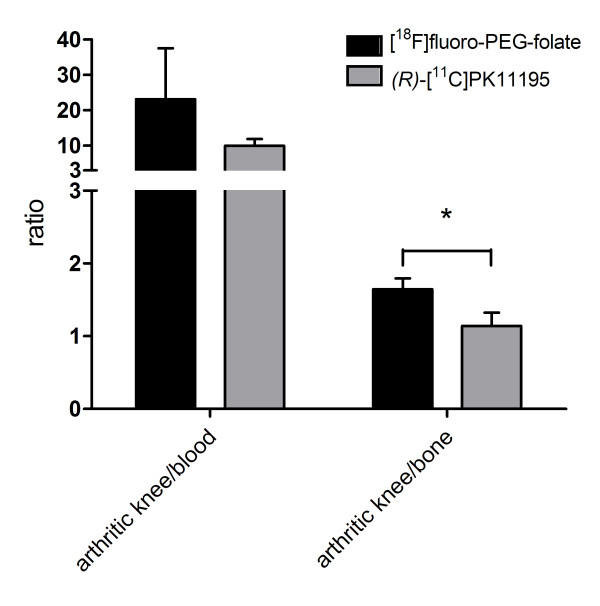
**Target-to-background ratios of [^18^F]fluoro-PEG-folate**. Arthritic knee-to-bone and arthritic knee-to-blood ratios of [^18^F]fluoro-PEG-folate (black bars) and *(R)-*[^11^C]PK11195 (grey bars) obtained from *ex vivo *tissue distribution studies. Results are presented as mean ± SD of four rats for [^18^F]fluoro-PEG-folate and five rats for *(R)-*[^11^C]PK11195. *= *P *<0.05

Tissue distribution of [^18^F]fluoro-PEG-folate was compared with that of the established macrophage targeting PET tracer *(R)*-[^11^C]PK11195. [^18^F]fluoro-PEG-folate cleared more rapidly from blood than *(R)-*[^11^C]PK11195, resulting in significantly lower blood levels of [^18^F]fluoro-PEG-folate than of *(R)-*[^11^C]PK11195 (0.02 ± 0.02 vs 0.08 ± 0.01, respectively; *P *<0.05). Consequently, mean arthritic knee-to-blood ratios of [^18^F]fluoro-PEG-folate were higher than those of *(R)-*[^11^C]PK11195 (23.1 ± 14.3 vs 10.0 ± 1.9, respectively) (Figure 6). Mean absolute uptake (%ID/gram) of [^18^F]fluoro-PEG-folate was lower than that of *(R)-*[^11^C]PK11195 in various tissues including arthritic knees (0.34 ± 0.08 vs 0.79 ± 0.05, respectively; *P *<0.05), contralateral knees (0.24 ± 0.08 vs 0.57 ± 0.05, respectively; *P *<0.05) and other internal organs, such as heart, lungs, spleen and gastrointestinal organs (data not shown).

Mean arthritic-to-contralateral knee uptake ratios of [^18^F]fluoro-PEG-folate (1.42 ± 0.15) and *(R)-*[^11^C]PK11195 (1.39 ± 0.10) were similar. Nevertheless, with peri-articular bone as the most relevant background tissue, mean arthritic-to-ipsilateral bone ratios of [^18^F]fluoro-PEG-folate (1.64 ± 0.15) were significantly higher than those of *(R)-*[^11^C]PK11195 (1.14 ± 0.18, *P *<0.05) (Figure 6).

### Blocking studies

After *in vivo *blockade of the FR with a ≥100-fold molar excess of glucosamine-folate, arthritis was no longer visualized on PET images (see Additional file 3, Figure S3B). In addition, a marked decrease in mean [^18^F]fluoro-PEG-folate tracer uptake (%ID/gram) in the arthritic knee (0.03 ± 0.02) was found in *ex vivo *tissue distribution studies (Figure 5), indicating specific binding of the tracer to FR. Finally, reduced accumulation of [^18^F]fluoro-PEG-folate was also observed in liver (0.06 ± 0.07) and spleen (0.02 ± 0.01), while uptake in kidneys (1.52 ± 0.81) and intestines (0.24 ± 0.31) was reduced but still appreciable.

### Effects of reducing plasma folate levels

Mean plasma serum levels in rats fed with folate deficient chow for 14 days decreased from 110 ± 21 to 11.0 ± 4.3 nM (that is, comparable with human physiological levels).

Mean tissue radioactivity (%ID/gram) found in arthritic knees of rats on folate deficient chow (0.52 ± 0.10) was increased compared with that of rats on normal chow (0.34 ± 0.08, *P *<0.05). Mean arthritic knee-to-bone ratios (1.38 ± 0.17) did not improve under folate deficient conditions, since uptake of the tracer in ipsilateral bone tissue increased in parallel.

## Discussion

In this study, the synthesis of the novel FR ligand [^18^F]fluoro-PEG-folate was described. In addition, initial *in vitro *and *in vivo *preclinical studies were performed that showed that this ligand was suitable as a PET tracer for imaging of arthritis.

The results of the *in vitro *binding studies showed high binding affinity of fluoro-PEG-folate to FR present on KB cells. KB cells, which overexpress FR-α, were used as a model system since folic acid and derivatives bind to both FR-α and FR-β (expressed on activated macrophages) with comparable affinity [36]. Accumulation of the tracer in arthritic knee joints of rats was shown by both *in vivo *PET scans and by *ex vivo *tissue distribution studies. Specific binding of [^18^F]fluoro-PEG-folate to FR in the macrophage-rich synovium was demonstrated by *in vivo *blocking studies.

As a tracer, [^18^F]fluoro-PEG-folate shows promise, as the lower peri-articular uptake in bone (marrow) and the faster clearance from blood compared with *(R)-*[^11^C]PK11195 provide improved contrast when imaging arthritic joints. This will especially be important for the imaging of subclinical disease activity.

The mBSA induced rat arthritis model is an established model of RA, which resembles human pathology of RA at the joint level [29]. A limitation of the rat model is the small size of the arthritic knee joints and, hence, a very small volume of inflamed synovia, which hampered exact determination of tracer uptake in synovial tissue. The spatial resolution and anatomical information provided by the PET scanner were not sufficient for exact definition of synovial ROIs. Instead, an ROI was drawn on top of the knee area, which also may include some peri-articular bone (marrow). In addition, isolation of pure synovium for *ex vivo *tissue distribution studies was not feasible. Instead, the knee joint as a whole, including some peri-articular soft tissue, was excised and prepared for analysis. Both absolute uptake, arthritic joint-to-bone (marrow) ratios and arthritic-to-contralateral knee ratios of [^18^F]fluoro-PEG-folate may, therefore, have been underestimated. Statistical significance of the differences between uptake (ratios) of [^18^F]fluoro-PEG-folate and *(R)-*[^11^C]PK11195, and between uptake in arthritic versus control joints was further reduced due to lack of statistical power related to the relatively small numbers of animals (*n *= 4 to 8) per group.

Relatively high [^18^F]fluoro-PEG-folate uptake was observed in the liver and spleen of rats with mBSA induced arthritis, which was largely abolished by FR receptor blockade with glucosamine-folate, suggesting specific receptor binding of the tracer. Conceivably, this observation may be explained by an increase of macrophage-like cells in the liver and spleen due to systemic immune activation as part of locally antigen induced arthritis [37,38]. Similarly, increased (specific) liver and spleen uptake of the folate conjugate EC20 was noted in rats with adjuvant induced arthritis compared to normal rats [20,39]. Further corroboration was found in tissue distribution studies, showing that uptake of [^18^F]fluoro-PEG-folate in the liver and spleen were respectively 3.8 and 2.2 times higher in arthritic than in normal rats (results not shown). Part of the liver and intestinal uptake of the novel folate tracer was, however, not blocked by glucosamine-folate, which could be due to the hepatobiliary secretion of the tracer. Likewise, [^18^F]fluoro-PEG-folate kidney uptake could be partly abolished under FR-blockade conditions, which was anticipated from folate receptor expression in proximal tubule cells, but leaving retention of the tracer in the kidney, possibly due to renal clearance. Additionally, non-specific tissue uptake of [^18^F]fluoro-PEG-folate or radiometabolite formation could also have influenced tissue distribution studies.

The ultimate value of [^18^F]fluoro-PEG-folate for imaging of RA has to be corroborated in human studies, which is readily achievable since the current method for the synthesis of [^18^F]fluoro-PEG-folate can easily be translated into a Good Manufacturing Practice compliant method yielding a clinical grade [^18^F]fluoro-PEG-folate radiotracer. Since human 5-Me-THF plasma and tissue levels are substantially lower compared to rat it is conceivable that absolute uptake of [^18^F]fluoro-PEG-folate in inflamed joints may be higher in humans. Nevertheless, simultaneously increased background uptake of [^18^F]fluoro-PEG-folate was shown in rats fed with folate deficient chow. Furthermore, it should be acknowledged that humans exhibit generally higher FR expression levels in the kidneys compared to rats [10], which could lead to higher accumulation of [^18^F]fluoro-PEG-folate in human patients than was found in our experiments performed in rats. Thus, it remains to be seen whether lower circulating 5-Me-THF plasma levels and generally higher FR expression tissue levels in humans set favorable conditions for [^18^F]fluoro-PEG-folate PET imaging of RA.

Currently, the single-photon emission computed tomography **(**SPECT) imaging agent ^99m^Tc-EC20 is the only FR-targeting radioligand that has been used for clinical applications in RA patients [22]. ^99m^Tc-EC20 was safely applied and targeted to inflamed joints. Although SPECT may be a cheaper alternative, PET imaging provides higher sensitivity and spatial resolution and allows signal quantification. Given the notion that the FR has also been recognized as an important target for (image guided) delivery of therapeutics, FR-based PET imaging could be exploited to select patients who would benefit from FR mediated targeting of activated macrophages by antifolates [12]. The folate antagonist methotrexate (MTX) is the most frequently prescribed disease modifying antirheumatic drug for RA, but side effects and resistance to MTX often occur [40]. MTX exhibits a relatively low binding affinity for FR, hence more selective targeting of folate antagonists to FR expressing cells in the synovium may be of value if therapy with MTX fails [12]. Other therapy strategies could include folate-immunoconjugates [41] and folate-conjugated vincristine [42].

Finally, imaging of macrophages with folate radiotracers can also be used for other diseases with macrophage activation [43,44]. For example, visualization of activated macrophages in atherosclerosis may be of value in patients at increased cardiovascular risk, which includes RA patients [45].

## Conclusions

In conclusion, highly sensitive arthritis imaging by PET and macrophage targeting using the novel [^18^F]fluoro-PEG-folate tracer was demonstrated in an mBSA induced arthritis model in rats. [^18^F]fluoro-PEG-folate shows higher contrast due to a lower background signal than the established, clinically available macrophage tracer *(R)-*[^11^C]PK11195. These promising results set the stage for proof of concept studies in RA patients.

## Abbreviations

3D: 3-dimensional; 5-Me-THF: 5-methyltetrahydrofolate; BSA: bovine serum albumin; FITC: fluorescein isothiocyanate; FR: folate receptor; IDID: image derived injected dose; mBSA: methylated bovine serum albumin; MTX: methotrexate; PBS: phosphate-buffered saline; PEG: polyethylene glycol; PET: positron emission tomography; RA: rheumatoid arthritis; ROI: region of interest; SD: standard deviation; SFB: succinylfluorobenzoate; SPECT: single-photon emission computed tomography; SUV: standardized uptake value

## Competing interests

PL owns stock and receives financial compensation from Endocyte Inc., a company that develops folate-targeted imaging and therapeutic agents. The other authors declare that they have no competing interests.

## Authors' contributions

YG made substantial contributions to acquisition, analysis and interpretation of the data and drafted the manuscript. KW made substantial contributions to acquisition, analysis and interpretation of the data and helped draft the manuscript. CM, GJ, AL and CL participated in the design of the study, made substantial contributions to interpretation of the data and critically revised the manuscript. AW coordinated the design and manufacturing of the PET tracers, helped draft of the manuscript and made substantial contributions to revision of the manuscript. MH helped draft the manuscript and made substantial contributions to the data analysis of the PET scans and revision of the manuscript. DS carried out the LC-MS/MS assay for determination of the plasma folate levels and helped with drafting the manuscript. SK and PL made substantial contributions to design of the study and revision of the manuscript. All authors read and approved the final manuscript.

## Supplementary Material

Additional file 1, Figure S1 Representative HPLC chromatograms of the purification and analysis of [^18^F]fluoro-PEG-folate. (**A**) Semi prep HPLC chromatogram of the purification of [^18^F]fluoro-PEG-folate; top: UV detection, bottom: radioactivity detection. The product elutes at 31 to 33 minutes, radiochemical yield, calculated from this chromatogram is 73%. The non-radioactive reference compound eluted at the same retention time as the radioactive compound (result not shown) (**B**) Analysis (quality control) of the final solution of [^18^F]fluoro-PEG-folate; top: UV detection, bottom: radioactivity detection. Radiochemical purity >99%. (**C**) Analysis (quality control) of the final solution of [^18^F]fluoro-PEG-folate, with the addition of reference compound; top: UV detection, bottom: radioactivity detection. The reference compound elutes at the same time as [^18^F]fluoro-PEG-folate and confirms the identity of [^18^F]fluoro-PEG-folate. Click here for file

Additional file 2, Figure S2 Uptake [^18^F]fluoro-PEG-folate in arthritic knee and blood. (**A**) Time-activity curves of [^18^F]fluoro-PEG-folate uptake in arthritic knee and blood. (**B**) Arthritic knee-to-blood ratio as function of time. Results are presented as mean ± SD of four arthritic rats. Click here for file

Additional file 3, Figure S3 [^18^F]fluoro-PEG-folate imaging of arthritic rats. [^18^F]fluoro-PEG-folate uptake in the right arthritic knee at (**A**) baseline (arrow) and (**B**) after blocking the FR with an excess dose of glucosamine-folate. In the latter case abolishment of the signal in the arthritic knee is evident. Click here for file
