# Supplementary figures and images for: Evaluation of the novel folate receptor ligand [18F]fluoro-PEG-folate for macrophage targeting in a rat model of arthritis
Source: Arthritis Res Ther. 2013 Mar 1;15(2):R37. doi: 10.1186/ar4191 (PMC3672671; doi:10.1186/ar4191)

## Slide 1
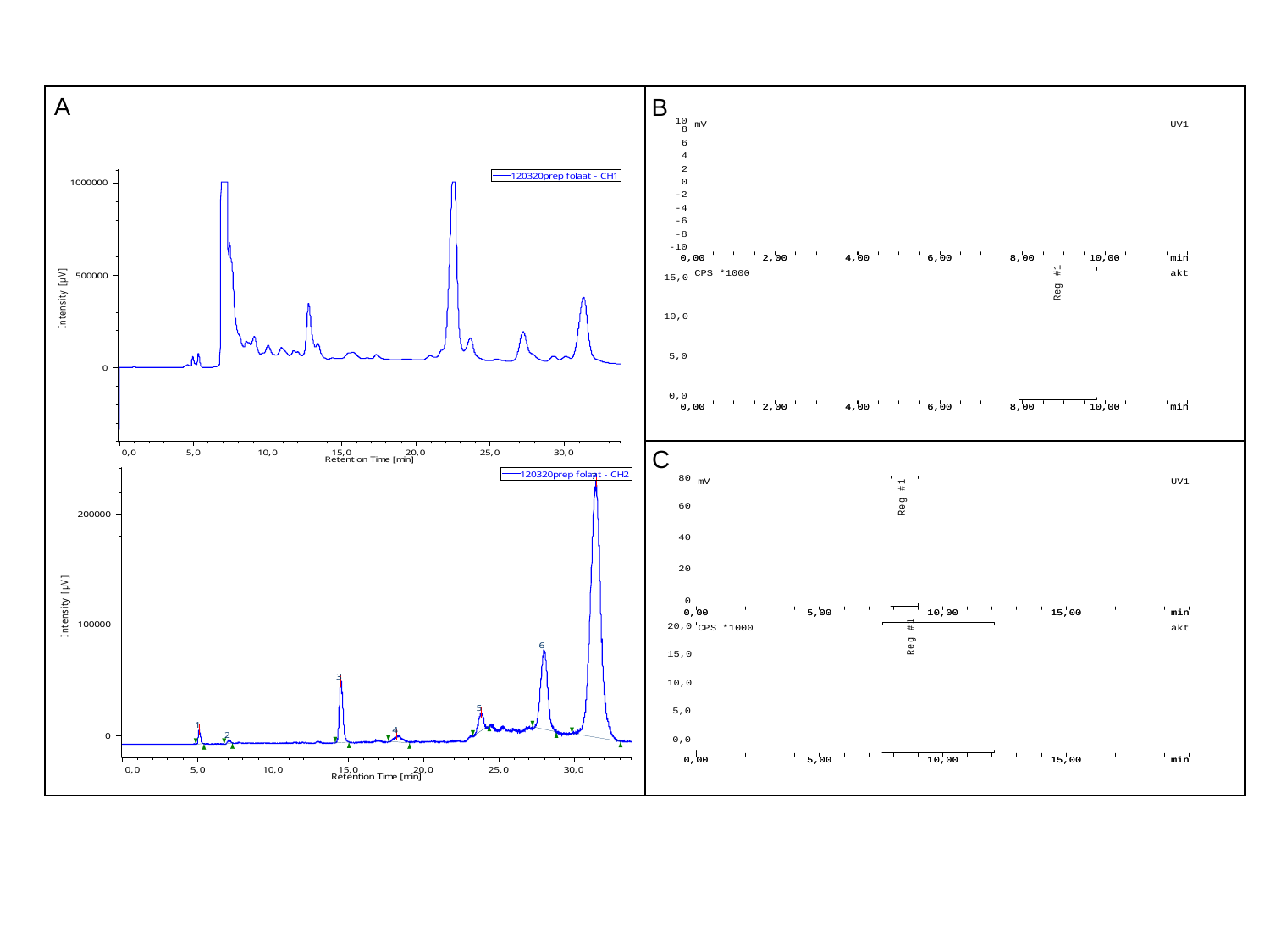

A
B
C

Supplement: Additional file 1, Figure S1 — Representative HPLC chromatograms of the purification and analysis of [18F]fluoro-PEG-folate. (A) Semi prep HPLC chromatogram of the purification of [18F]fluoro-PEG-folate; top: UV detection, bottom: radioactivity detection. The product elutes at 31 to 33 minutes, radiochemical yield, calculated from this chromatogram is 73%. The non-radioactive reference compound eluted at the same retention time as the radioactive compound (result not shown) (B) Analysis (quality control) of the final solution of [18F]fluoro-PEG-folate; top: UV detection, bottom: radioactivity detection. Radiochemical purity >99%. (C) Analysis (quality control) of the final solution of [18F]fluoro-PEG-folate, with the addition of reference compound; top: UV detection, bottom: radioactivity detection. The reference compound elutes at the same time as [18F]fluoro-PEG-folate and confirms the identity of [18F]fluoro-PEG-folate. [file ar4191-S1.PPT]

## Slide 1
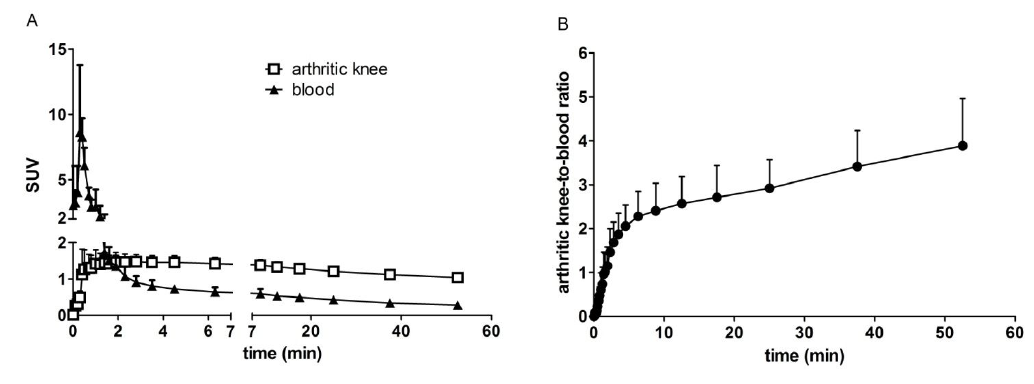

Supplement: Additional file 2, Figure S2 — Uptake [18F]fluoro-PEG-folate in arthritic knee and blood. (A) Time-activity curves of [18F]fluoro-PEG-folate uptake in arthritic knee and blood. (B) Arthritic knee-to-blood ratio as function of time. Results are presented as mean ± SD of four arthritic rats. [file ar4191-S2.PPT]

## Slide 1
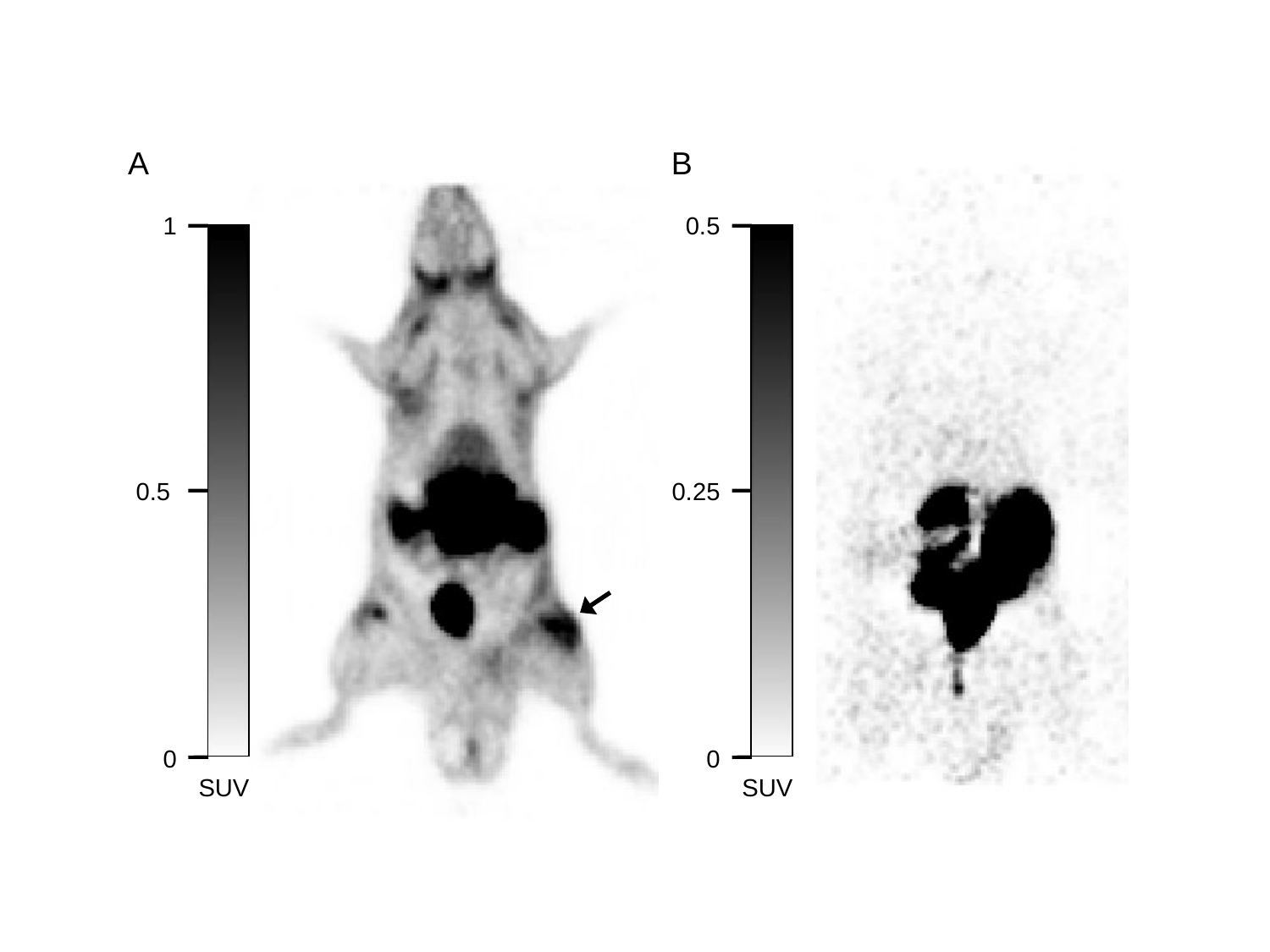

A
B
1
0.5
0.5
0.25
0
0
SUV
SUV

Supplement: Additional file 3, Figure S3 — [18F]fluoro-PEG-folate imaging of arthritic rats. [18F]fluoro-PEG-folate uptake in the right arthritic knee at (A) baseline (arrow) and (B) after blocking the FR with an excess dose of glucosamine-folate. In the latter case abolishment of the signal in the arthritic knee is evident. [file ar4191-S3.PPT]
